# Epidemiology of Emergency Medical Services Activations for Sport-Related Injuries in the United States

**DOI:** 10.7759/cureus.27403

**Published:** 2022-07-28

**Authors:** Rebecca M Hirschhorn, Zachary Y Kerr, James M Mensch, Robert A Huggins, Thomas P Dompier, Caroline Rudisill, Susan W Yeargin

**Affiliations:** 1 School of Kinesiology, Louisiana State University, Baton Rouge, USA; 2 Department of Exercise and Sport Science, University of North Carolina at Chapel Hill, Chapel Hill, USA; 3 Department of Exercise Science, Arnold School of Public Health - University of South Carolina, Columbia, USA; 4 Department of Kinesiology, University of Connecticut, Storrs, USA; 5 School of Health Professions, Lebanon Valley College, Annville, USA; 6 Health Promotion, Education and Behavior, Arnold School of Public Health - University of South Carolina, Columbia, USA

**Keywords:** sports, hospital, emergency service, athletes, ambulances

## Abstract

Background

Literature examining emergency medical services (EMS) activations for sport-related injuries is limited to the pediatric, high school, and collegiate student-athlete populations, excluding older individuals and recreational athletes. The purpose of this study was to examine EMS activations for sport-related injuries using the National EMS Information System Database from 2017-2018.

Methods

Data were obtained using the National EMS Information System Database from 2017-2018. EMS activations were limited to 9-1-1 responses for individuals aged 3-99 who sustained a sports-related injury. Independent variables included patient age group: pediatric (<18 years old) vs. adult (≥18 years old). Dependent variables were patient age, gender, and chief complaint anatomic location. Frequencies and proportions were calculated for each variable. Injury proportion ratios (IPRs) with 95% confidence intervals were calculated to compare chief complaint anatomic location by age group.

Results

There were 71,322 sport-related injuries. Patients were 36.6±22.9 years and most (58.1%, n=41,132) were male. Adults had higher proportions of injuries affecting the abdomen (IPR: 2.05, 95%CI: 1.83, 2.31), chest (IPR: 1.90, 95%CI: 1.75, 2.05), general/global (IPR: 1.54, 95%CI: 1.50, 1.58), and genitalia (IPR: 2.40, 95%CI: 1.39, 4.15), and lower proportions of injuries affecting the back (IPR: 0.55, 95%CI: 0.50, 0.60), lower extremity (IPR: 0.63, 95%CI: 0.60, 0.65), upper extremity (IPR: 0.50, 95%CI: 0.47, 0.53), head (IPR: 0.73, 95%CI: 0.70, 0.77), and neck (IPR: 0.18, 95%CI: 0.16, 0.20) compared to pediatric patients.

Conclusion

Injuries sustained differed between adult and pediatric patients, indicating sport-related emergencies may change across the lifespan. General/global chief complaints likely indicate sport-related injuries affecting multiple anatomic locations and organ systems. Stakeholders planning large or high-risk athletic events should consider arranging standby or dedicated advanced life support units for their events.

## Introduction

Emergency department (ED) visits resulting from sports participation are estimated at 76.1 visits per 100,000 population in the United States [1]. The number of sports-related injuries presented to EDs increased by over 10,000 annually in the decade leading up to 2013 [2]. Football, basketball, soccer, and baseball account for significant proportions of sports-related injuries presented to EDs [3-5]. However, these studies did not examine whether or not the patients arrived at the ED via emergency medical services (EMS) or another method of transportation, an essential indicator of emergency resource utilization.

Severe and rare injuries have accounted for 3-15% of sport-related injuries in collegiate and high school athletics [6-8]. During the 2018/19 academic year, 80 catastrophic injuries occurred among high school and collegiate athletes directly attributed to sports participation, 25 of which were fatal [9]. Approximately 14% of life-threatening injuries among pediatric patients are attributable to sports activities, equating to over 92,000 life-threatening sport-related injuries presenting to EDs annually [10]. Due to the nature of severe and catastrophic injuries, transportation by EMS is likely warranted in a variety of sports settings; however, the utilization of EMS was not studied. 

An EMS activation is when an EMS response is initiated when an individual calls 9-1-1. Among children, 11-22% of EMS activations originate from places of recreation or sport [11,12]. One study specifically examined pediatric EMS activations to places of recreation and sport using data collected by EMS agencies [12]; however, only location criteria were used to identify sports-related injuries while mechanism or cause of injury was not considered. Approximately 7% of pediatric patients presenting to the ED for a sport-related injury arrive via ambulance [3]. When surveyed, most high schools and collegiate institutions indicated they activated EMS one to two times per year, with some activating EMS six or more times [13]. This study was limited to football, indicating EMS activations are probably greater per year when considering all sports. Literature has primarily focused on pediatric sport-related injuries that occurred during organized sport and has left out adult athletic populations and other recreational sports settings.

Not all EMS activations result in treatment and transportation of the patient, which can occur when a patient refuses care or transportation by EMS or when the EMS providers can treat in place without transporting the patient to the ED. Between 49% and 89% of EMS activations result in transportation to EDs by EMS providers [11,14]. Procedures performed by EMS have been examined in both children and adults [11,15-18] but are not specific to sport-related injuries. Children have received an advanced level of care in approximately 15% of transports [11,15]. On average, at least one procedure was performed per EMS response, with monitoring occurring more often than therapeutic and critical advanced life support (ALS) procedures [17]. Understanding the level of care and type of procedures performed by EMS for sport-related injuries can help inform medical coverage decisions for athletic events (e.g., standby versus dedicated unit and level of care of the unit dispatched) and relevant EMS protocols. Additionally, findings may identify sport-related emergency scenarios where additional training may be warranted for first responders and EMS providers.

Emergency medical services are a critical link between the place of injury and the ED when sport-related emergencies occur. Current literature examining sport-related injuries transported by EMS is focused on the high school, and collegiate settings [13,19], leaving a broader understanding of sport-related emergencies unknown as sports participation occurs in a variety of settings by people of all ages. Several studies have examined sport-related injuries among pediatric patients presenting to EDs in the United States (US) [1-4,20], but research on the adult population is sparse [4,5]. The National EMS Information System (NEMSIS), a standardized EMS database, presents an opportunity to examine sport-related injuries from the perspective of EMS across the US. This study aimed to describe EMS activations for sport-related injuries using the NEMSIS. A secondary purpose was to compare pediatric (<18 years old) and adult (≥ 18 years old) injuries. It was hypothesized that there would be differences in the types of injuries encountered by EMS between the adult and pediatric populations. This article was previously presented as an e-poster at the Virtual National Athletic Trainers' Association Clinical Symposia & AT Expo from June 22 - October 18, 2021. 

## Materials and methods

Study design

A descriptive epidemiological design was conducted utilizing data from the NEMSIS for the 2017-2018 calendar years. These years were chosen because the NEMSIS began using ICD-10-CM codes in 2017. Emergency medical services activations included in this study were limited to 9-1-1 responses for a sport-related injury occurring in individuals aged 3 to 99. This range was selected due to the age of 3 years being the typical age at which children begin to participate in formal sporting activities, and 99 years being the highest defined upper age limit for the world master's athletics [21].

Study procedures

A data request was submitted to the NEMSIS for the 2017 and 2018 Public-Release Datasets. The NEMSIS collects data voluntarily reported by participating EMS agencies across the US. For each EMS activation, the EMS provider completes a patient care report. Emergency medical services agencies, at minimum, must include required national and state-level elements. The 2017 Public-Release Research Dataset included over 7.9 million EMS activations from 4,016 EMS agencies across 35 states and territories. The 2018 Public-Release Research Dataset included over 22.5 million EMS activations from 9,599 EMS agencies across 43 states and territories. The NEMSIS Technical Assistance Center checked data from participating agencies for completeness, logical consistency and formatting, and quality assurance.

**Figure 1:**
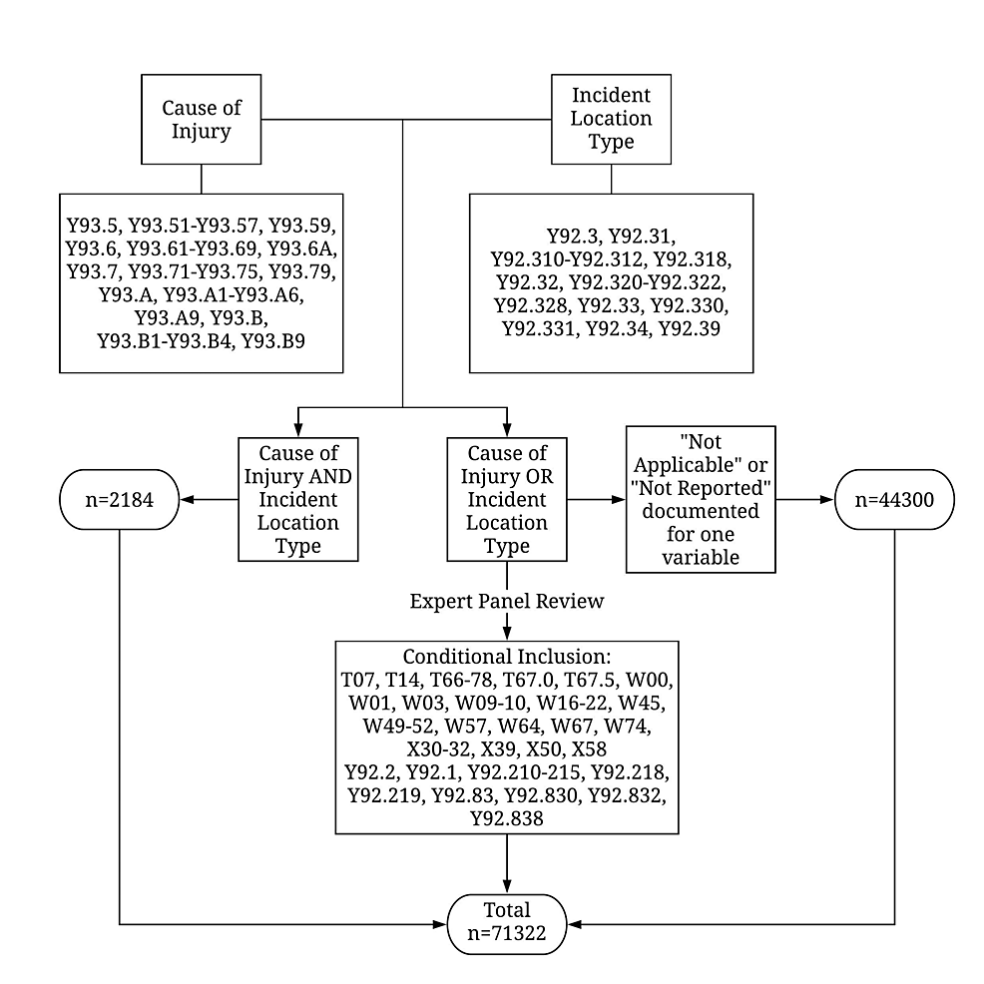
Inclusion criteria for identifying EMS activations for sport-related injuries

A sport-related injury was operationally defined as an EMS activation within the NEMSIS based on incident location and cause of injury. The ICD-10-CM codes were selected within the patient care report as determined by the EMS provider. Second, those resulting cases in which the cause of injury or incident location type were met were then reviewed by a panel of sports injury experts to confirm that they were likely due to sports participation. Those determined to be sports-related injuries were retained in the dataset (Figure 1).

Variables of interest

The NEMSIS variables of interest in this study were: level of care of this unit, patient gender, patient race, patient age, CMS service level, incident location type, chief complaint anatomic location, chief complaint organ system, provider's primary impression, cause of injury, procedures, incident/patient disposition, US census region, US census division, and urbanicity. Patient age (in years), US census division, US census region, and urbanicity were calculated and provided by the NEMSIS but not directly entered by the EMS provider completing the patient care report.

Statistical analysis

Data were analyzed using SAS® software (Version 9.4, SAS Institute Inc., Cary, NC, USA). Frequencies and proportions were calculated for all variables. Mean, and standard deviation was calculated for patient age. The age groups "pediatric" (<18 years old) and "adult" (≥18 years old) were created for comparison of chief complaint anatomic locations and organ systems. Injury proportion ratios (IPRs) with 95% confidence intervals (95%CIs) were calculated to compare chief complaint variables by age group. Injury proportion ratio confidence intervals excluding 1.00 were considered statistically significant. Cases in which a required variable was coded as “not applicable” or “not recorded” by the NEMSIS were recorded as “missing” and excluded from analyses. 

All data received by the current study's research team were de-identified prior to analysis. This study was considered exempt by the University of South Carolina Institutional Review Board (Pro00095944, 12/16/2019).

## Results

Overall, 71,322 EMS activations for sport-related injuries were identified (2017: 0.3%, n=20,903/7,907,829; 2018: 0.2%, n=50,419/22,532,890). The average patient age was 36.6±22.9 years. Most patients were white (70.0%, n=19,038/27,204) and male (58.1%, n=41,132/70,780; Table 1). The West US Census Region contributed the largest proportion of EMS activations (39.2%, n=27,986/71,322) and most occurred in Urban areas (86.2%, n=59,127/68,601). The patient was treated by EMS in 87.9% (n=62,677) of EMS activations (Table 2). In 4.5% of cases (n=3,240) the patient was evaluated and determined to not need treatment or transport by EMS and in 5.6% of cases (n=4,011) the patient refused evaluation/care and transport by EMS. Most units were staffed as ALS-Paramedic (80.0%, n=57,058), followed by BLS-Basic/EMT (13.0%, n=9,242). Five percent (n=3,564) of responding units were staffed at the Advanced EMT or Intermediate level.

**Table 1:** Patient demographics of EMS activations for sport-related injuries from 2017-2018 (n=71,322)

Variable	n (%)
Patient Age	
Pediatric (≤18)	20010 (28.1)
Adult (>18)	51312 (71.9)
Average (SD); Range	36.6 (22.9); 3-99
Patient Gender^1^	
Female	29618 (41.9)
Male	41132 (58.1)
Unknown (Unable to Determine)	30 (0.0)
Patient Race^2^	
American Indian or Alaska Native	329 (1.2)
Asian	486 (1.8)
Black or African American	4356 (16.0)
Hispanic or Latino	2837 (10.4)
Native Hawaiian or Other Pacific Islander	158 (0.6)
White	19038 (70.0)
US Census Division	
East North Central	5574 (7.8)
East South Central	4231 (5.9)
Middle Atlantic	3546 (5.0)
Mountain	13952 (19.6)
New England	2943 (4.1)
Pacific	14034 (19.7)
South Atlantic	16591 (23.3)
Territories	136 (0.2)
West North Central	5009 (7.0)
West South Central	5306 (7.4)
US Census Region	
Island Areas	136 (0.2)
Midwest	10583 (14.8)
Northeast	6489 (9.1)
South	26128 (36.6)
West	27986 (39.2)
Urbanicity^3^	
Rural	5005 (7.3)
Suburban	3146 (4.6)
Urban	59127 (86.2)
Wilderness	1323 (1.9)
Data originated from the National Emergency Medical Services Information System (2017-2018) ^1^ Patient gender was not documented in 542 cases ^2^ Patient race was not documented in 44118 cases ^3^ Urbanicity was not reported in 2721 cases	

**Table 2:** Incident/patient disposition of EMS activations for sport-related injuries (n=71.322)

Incident/Patient Disposition	n (%)
Assist^1^	827 (1.2)
Canceled^2^	96 (0.1)
Patient Dead at Scene^3^	168 (0.2)
Patient Evaluated, No Treatment/Transport Required	3240 (4.5)
Patient Refused Evaluation/Care (With Transport)	22 (0.0)
Patient Refused Evaluation/Care (Without Transport)	4011 (5.6)
Patient Treated, Released (AMA)	7095 (10.0)
Patient Treated, Released (Per Protocol)	2467 (3.5)
Patient Treated, Transferred Care to Another EMS Unit^4^	5056 (7.1)
Patient Treated, Transported by this EMS Unit^5^	47385 (66.4)
Patient Treated, Transported by Law Enforcement	110 (0.2)
Patient Treated, Transported by Private Vehicle	564 (0.8)
Standby^5^	281 (0.4)
Abbreviations: AMA, against medical advice; EMS, emergency medical services Data originated from the National Emergency Medical Services Information System (2017-2018) ^1^ Includes agency, public, and unit assistance ^2^ Includes canceled prior to arrival at scene, no patient contact, and no patient found ^3^Includes no resuscitation with and without transport and resuscitation attempt with and without transport ^4^ Considered “treated and transported by EMS” (n=52,441, 73.5%) ^5^ Includes no services or supported provided and public safety, fire, or EMS operational support	

Incident location type

Incident location type was missing in 19 cases (total n=71,303). The most frequent incident location types that EMS responded to were “gymnasium” (36.7%, n=26,196) and “sports and athletics area” (27.8%, n=19,821). Other common locations included “athletic field” (12.7%, 9,087), “athletic court” (6.7%, n=4,751), and “swimming pool (public)” (5.3%, n=3,798). Schools were documented as the incident location in 2.7% (n=1,950) of EMS activations for sport-related injuries. Sport-specific athletic areas were infrequently selected (3.4%, n=2408).

Chief complaint anatomic location and organ system

Chief complaint anatomic location was missing in 20,006 cases (total n=51,316). Chief complaints that were “general/global” were the most prevalent, accounting for 46.9% (n=24,075) of EMS activations, followed by lower extremity (14.3%, n=7,351) and head (13.2%, n=6,784). Adults had higher proportions of injuries affecting the abdomen (IPR: 2.05, 95%CI: 1.83, 2.31), chest (IPR: 1.90, 95%CI: 1.75, 2.05), general/global (IPR: 1.54, 95%CI: 1.50, 1.58), and genitalia (IPR: 2.40, 95%CI: 1.39, 4.15) compared to pediatric patients (Table 3). Conversely, adults had lower proportions of injuries affecting the back (IPR: 0.55, 95%CI: 0.50, 0.60), lower extremity (IPR: 0.63, 95%CI: 0.60, 0.65), upper extremity (IPR: 0.50, 95%CI: 0.47, 0.53), head (IPR: 0.73, 95%CI: 0.70, 0.77), and neck (IPR: 0.18, 95%CI: 0.16, 0.20) compared to pediatric patients.

**Table 3:** Chief complaint anatomic location by age group for EMS activations of sport-related injuries

	Age Group (n,%)			
Anatomic Location	Adult (≥18)	Pediatric (<18)	Total	Adult Versus Pediatric Injury Proportion Ratio (95% CI)
Abdomen	1658 (4.5)	327 (2.2)	1985 (3.9)	2.05 (1.83, 2.31)^1^
Back	942 (2.6)	695 (4.7)	1637 (3.2)	0.55 (0.50, 0.60)^1^
Chest	3314 (9.1)	708 (4.8)	4022 (7.8)	1.90 (1.75, 2.05)^1^
Lower Extremity	4468 (12.2)	2883 (19.5)	7351 (14.3)	0.63 (0.60, 0.65)^1^
Upper Extremity	2188 (6.0)	1788 (12.1)	3976 (7.7)	0.50 (0.47, 0.53)^1^
General/ Global	19065 (52.2)	5010 (33.9)	24075 (46.9)	1.54 (1.50, 1.58)^1^
Genitalia	89 (0.2)	15 (0.1)	104 (0.2)	2.40 (1.39, 4.15)^1^
Head	4373 (12.0)	2411 (16.3)	6784 (13.2)	0.73 (0.70, 0.77)^1^
Neck	430 (1.2)	952 (6.4)	1382 (2.7)	0.18 (0.16, 0.20)^1^
Total	36527 (100.0)	14789 (100.0)	51316 (100.0)^2^	NA
Abbreviations: CI, confidence interval; NA, not applicable				
Data originated from the National Emergency Medical Services Information System (2017-2018) ^1^ Statistically significant 95% CI ^2 ^Chief complaint anatomic location was missing in n=20,006 cases and excluded from analysis				

Chief complaint organ system was missing in 18,549 cases (total n=52,773). “Global/general” was also the most prevalent organ system affected, accounting for 38.9% of EMS activations (n=20,543). The next most common organ systems affected were musculoskeletal/skin (28.8%, n=15,208) and central nervous system/neurological (14.6%, n=7,703). Adults had higher proportions of injuries involving the behavioral/psychiatric (IPR: 2.36, 95%CI: 2.00, 2.79), cardiovascular (IPR: 5.99, 95%CI: 5.21, 6.88), central nervous system/neurological (IPR: 1.25, 95%CI: 1.19, 1.31), endocrine/metabolic (IPR: 2.13, 95%CI: 1.84, 2.47), gastrointestinal (IPR: 2.45, 95%CI: 2.12, 2.82), global/general (IPR: 1.23, 95%CI: 1.19, 1.26), and reproductive (IPR: 5.01, 95%CI: 2.63, 9.54) systems compared to the pediatric population (Table 4). Conversely, adults had lower proportions of injuries affecting the musculoskeletal/skin (IPR: 0.50, 95%CI: 0.49, 0.52) and pulmonary (IPR: 0.81, 95%CI: 0.73, 0.89) systems compared to pediatric patients.

**Table 4:** Chief complaint organ system by age group for EMS activations of sport-related injuries

	Age Group (n,%)			
Organ System	Adult (≥18)	Pediatric (<18)	Total	Adult Versus Pediatric Injury Proportion Ratio (95% CI)
Behavioral/ Psychiatric	935 (2.5)	159 (1.1)	1094 (2.1)	2.36 (2.00, 2.79)^1^
Cardiovascular	3122 (8.3)	209 (1.4)	3331 (6.3)	5.99 (5.21, 6.88)^1^
CNS/ Neurological	5832 (15.5)	1871 (12.4)	7703 (14.6)	1.25 (1.19, 1.31)^1^
Endocrine/ Metabolic	1101 (2.9)	207 (1.4)	1308 (2.5)	2.13 (1.84, 2.47)^1^
Gastrointestinal	1299 (3.4)	213 (1.4)	1512 (2.9)	2.45 (2.12, 2.82)^1^
Global/ General	15479 (41.1)	5064 (33.5)	20543 (38.9)	1.23 (1.19, 1.26)^1^
Lymphatic/ Immune	147 (0.4)	52 (0.3)	199 (0.4)	1.13 (0.83, 1.55)
Musculoskeletal/ Skin	8455 (22.4)	6753 (44.7)	15208 (28.8)	0.50 (0.49, 0.52)^1^
Reproductive	125 (0.3)	10 (0.1)	135 (0.3)	5.01 (2.63, 9.54)^1^
Pulmonary	1145 (3.0)	568 (3.8)	1713 (3.2)	0.81 (0.73, 0.89)^1^
Renal	27 (0.1)	0 (0.0)	27 (0.1)	NA
Total	37667 (100.0)	15106 (100.0)	52773 (100.0)^2^	NA
Abbreviations: CI, confidence interval; NA, not applicable; CNS, central nervous system				
Data originated from the National Emergency Medical Services Information System (2017-2018) ^1^ Statistically significant 95% CI ^2^ Chief complaint organ system was missing in n=18,549 cases and excluded from analysis				

Provider impression

Provider primary impression was missing in 5,224 cases (total n=66,098). The most frequent primary impression selected by EMS providers was unspecified injury (16.9%, n=11,175), followed by syncope and collapse (8.0%, n=5,291) and acute pain (4.5%, n=2,947). Other common primary impressions included unspecified injury of the head (3.5%, n=2,339), weakness (3.2%, n=2,100), and altered mental status (2.5%, n=1,664; Table 5).

**Table 5:** Most common primary impressions within EMS activations for sport-related injuries (n=66,098)

Provider Primary Impression^1^	n (%)
Acute Pain, Not Elsewhere Classified	2947 (4.5)
Acute Respiratory Distress Syndrome	832 (1.3)
Alcohol Use, Unspecified	1639 (2.5)
Altered Mental Status, Unspecified	1664 (2.5)
Angina Pectoris, Unspecified	740 (1.1)
Back Pain, Not Otherwise Specified	999 (1.5)
Cardiac Arrhythmia, Unspecified	758 (1.2)
Dehydration	1148 (1.7)
Dizziness and Giddiness	1201 (1.8)
Encounter for General Examination Without Complaint, Suspected or Reported Diagnosis	695 (1.1)
Epilepsy, Unspecified, Not Intractable, Without Status Epilepticus	1508 (2.3)
Headache	897 (1.4)
Heat Exhaustion, Unspecified	1326 (2.0)
Injury, Unspecified	11175 (16.9)
Mental Disorder, Not Otherwise Specified	873 (1.3)
Other^2^	25177 (37.2)
Pain, Unspecified	826 (1.3)
Syncope and Collapse	5291 (8.0)
Unspecified Convulsions	843 (1.3)
Unspecified Injury of Head	2339 (3.5)
Unspecified Injury of Lower Leg	1120 (1.7)
Weakness	2100 (3.2)
Data originated from the National Emergency Medical Services Information System (2017-2018) ^1 ^Provider primary impression was missing in 5224 cases ^2^ All other provider primary impressions accounted for ≤1.0% individually	

Cause of injury

No cause of injury was provided for 44,315 cases, accounting for over 62% of EMS activations (total n=27,007). The most frequently documented causes of injury were "unspecified fall" (22.0%, n=5,947) and "fall on the same level from slipping, tripping, and stumbling" (16.8%, n=5,806). Sport activities played individually accounted for 8.7% (n=2,346) of EMS activations and those played as a team/group accounted for 6.6% (n=1,775). Other activities that accounted for ≥5% of EMS activations included "striking against or struck by sports equipment" (5.3%, n=1,444) and "accidental hit, strike, kick, twist, bite, or scratch by another person" (5.0%, n=1,356).

Procedures

**Figure 2:**
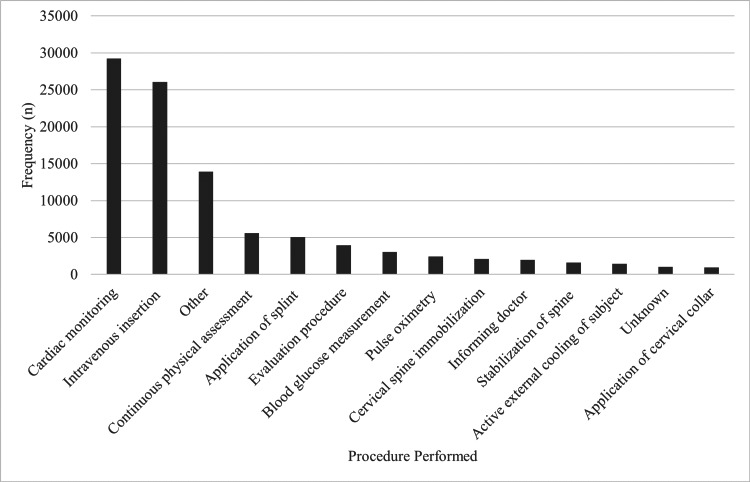
Most common procedures performed by EMS providers (n=98,325)

In total, 98,325 procedures were documented in this study, resulting in an average of 1.6 procedures performed per EMS activation in which the patient was treated by EMS (98,325/62,677). Note that procedures recorded as vital signs in the patient care report (i.e., blood pressure, respiration, heart rate, pulse oximetry) are not required to be separately documented in the procedure section of the report. The most prevalent procedures were cardiac monitoring (29.7%, n=29,220), intravenous insertion (26.5%, n=26,074)), and continuous physical assessment (5.7%, n=5,594; Figure 2). Other notable procedures performed for sport-related injuries included splint application (5.2%, n=5,081), cervical spine immobilization (2.1%, n=2,079), and active external cooling (1.4%, n=1,419). External ventricular defibrillation (0.7%, n=695) and cardiopulmonary resuscitation (0.6%, n=605) were also documented in this sample. The category "Other" is the sum of all other procedures performed that accounted for <1.0% each of the total procedures performed.

## Discussion

Overall, sport-related injuries accounted for a small proportion of all EMS activations within the NEMSIS from 2017-2018. Although previous research has also found that sport-related injuries transported by EMS typically represent a small percentage of overall injuries [19], it is essential to emphasize that such injuries account for significant proportions of life-threatening injuries treated in EDs [10]. This study demonstrates the broad utilization of EMS for sport-related injuries. It is the first to examine EMS activations due to sport-related injuries using a national database and identified sport-related injuries using cause and location type variables. We believe our sample excluded injuries that were not the result of sports participation despite occurring at an athletic venue (e.g., spectator).

Nearly 75% of the EMS activations in this study resulted in treatment and transport by an EMS unit. The proportion of EMS activations treated and transported within published NEMSIS data has varied from 49% to 59% in previous years [14]. Our findings indicate that a more significant proportion of individuals who sustained a sport-related injury accepted treatment and transport by EMS compared to the general population. This may be indicative of increased medical necessity for sport-related injuries.

Patient characteristics

This study included pediatric and adult patients, which contrasts with previous literature that has focused solely on the pediatric population [12] or was not specific to sport-related injuries [11,15,22,23]. Over two-thirds of EMS activations were for adults. The utilization of EMS has been shown to increase with age among the general population [24,25]. More individuals aged 15 to 24 participate in athletic activities than older age groups [26]. Our findings demonstrate that EMS activations for sport-related injuries are not limited to any specific age group. Planning for sport-related emergencies should not be limited to organized sports in younger populations and include settings that attract older age groups (e.g., club/recreational league sports).

Males accounted for most EMS activations, which was expected considering males have consistently accounted for more ED visits [1,3,5,20], EMS utilization [12,15], and participate in sports more frequently than females [26]. Males have also sustained more catastrophic sports injuries than females; however, this is likely because most resulted from football participation, a predominately male sport [9]. Stakeholders should not prioritize emergency coverage for events solely based on participant sex or gender. 

Incident location type

Most EMS activations in this study occurred in sports and athletic areas, as opposed to schools or other recreational areas. The current version of the NEMSIS data allowed for more specific locations to be selected by EMS providers compared to general categories' previous versions [11,12,23]. Sports are a leading factor in school-based EMS activations among the pediatric population [27]. In this study, locations such as schools or colleges/universities were not frequently documented; however, it was impossible to distinguish school-based athletics areas from non-school-based athletics areas based on the information provided. Sports and athletics include athletic fields, recreational areas, public and private swimming pools, and indoor and outdoor courts. Facilities hosting athletic activities should consider having an on-site medical professional trained in acute injury management, such as an athletic trainer. Each location comes with its unique challenges for EMS response (e.g., locked gates, stairs); thus, it is essential for organizations overseeing these areas to develop detailed access plans. It is also recommended to conduct a walk-through with the local EMS agency to ensure easy access for the responding EMS units.

Chief complaint anatomic location and organ system

This study's most frequently documented chief complaint anatomic locations were general/global, lower extremity, and head. The general/global category may include more systemic conditions (e.g., exertional heat illness, diabetic emergency, neurological conditions). This study's most frequently documented organ systems were general/global, musculoskeletal/skin, and central nervous system/neurological. Emergency medical services providers should have a high index of suspicion if the injury occurred to the head, neck, or torso, as these are most likely to be life-threatening [9,10]. Head and face injuries have frequently resulted in EMS transportation among high school and collegiate athletes [19]. Sport-related systemic injuries involving the pelvis, abdomen, chest, leg, hip, head, and back have increased the risk of hospital admission [5], indicating increased injury severity and the need for advanced medical care. In these cases, expedient transport to the ED is crucial due to the inability to rule out potentially life-threatening injuries without diagnostic imaging in the prehospital setting and the potential for rapid deterioration during transportation.

There were significant differences between pediatric and adult populations for every anatomic location and organ system except for lymphatic/immune. Age-related muscular, cardiovascular, and pulmonary function declines may contribute to the differences in adult sport-related injuries [28]. Underlying medical conditions, such as cardiovascular disease or diabetes, may also affect injury risk and clinical presentation in adults. Future research examining sport-related injuries in the adult population beyond collegiate athletics is warranted to understand how aging affects injury risk and subsequent EMS and ED utilization. Additionally, EMS providers should conduct a thorough evaluation, paying particular attention to previous medical history since it is unknown if other conditions may have contributed to or resulted from the sport-related injury.

Provider impression

The most common provider impressions documented in this study - injury, syncope and collapse, acute pain - are consistent with previous literature examining sport-related EMS activations and the general population [12,17,23]. Strains, sprains, fractures, concussions, and contusions are common sport-related injury diagnoses that have resulted in transportation by EMS [19] and could align with several provider impressions relating to injury types and pain in this study. Additionally, sport-related injuries considered systemic or affecting the abdomen, chest, and head are at an increased relative risk of being admitted to the hospital, indicating increased injury severity requiring advanced care [5]. Provider impressions are largely based on the subjective symptoms reported by the patient, mechanism of injury or onset of illness, and any visual signs noticed by the EMS provider, as there are limited diagnostic capabilities (e.g., glucometers, cardiac monitors) available in the prehospital setting. While EMS data provides valuable insight into sport-related injuries from the EMS provider's perspective, the accuracy of the provider impressions relative to the diagnoses determined in the ED is unknown. The ability of EMS agencies to link their electronic patient care reports to the hospital's electronic medical records would serve as a valuable quality improvement tool and identify where additional training or protocol modifications may be needed.

Cause of injury

Falls contributed to the most EMS activations in this study. In sports, falls can occur due to slipping or tripping on wet or uneven surfaces, improperly landing from a jump, or from elevated pieces of athletic equipment. Facilities should inspect playing surfaces for potential safety hazards such as puddles, protruding sprinklers, or uncovered grates. Falls are a common cause of injury resulting in EMS use in general [11,12,22,23,27]. A cause of injury was not documented in a large proportion of data in this study. Emergency medical services providers should include the cause or mechanism of injury in their documentation, as these data can provide insight into high-risk activities that may warrant rule changes in sports and evaluate the effectiveness of changes that have been implemented.

While the EMS activations in this study could not be analyzed by sport, participation in football, basketball, soccer, track and field, wrestling, baseball, ice hockey, and gymnastics frequently result in severe, rare, and catastrophic injuries including fatalities [6-9]. Standby EMS coverage is warranted for most sports, minimally at high school and college events [19]. Large-scale tournaments for younger and older athletes within these high-risk sports may also warrant standby coverage, especially if an on-site medical professional is not present.

Procedures

Obtaining intravenous access and cardiac monitoring (3- or 12-lead electrocardiograms), both of which are advanced skills, were the most frequently performed procedures in this study. Advanced life support level procedures are not commonly performed when looking at overall EMS activations in a population not specific to a medical condition [11,15,16,18]. Sudden cardiac arrest and other cardiac conditions account for nearly 50% of catastrophic sports-related injuries in the high school and collegiate populations [29]. Not all states require coaches of athletic teams to be certified in CPR and AED use. Athletic trainers are required to maintain BLS certification; however, only two-thirds of secondary schools have access to an athletic trainer [30], and it is unknown what proportion of recreational leagues or non-interscholastic sports have access to an athletic trainer. Therefore, the level of medical care available at sporting events is essential for EMS agencies to consider when notified about these events. When staffing a unit for standby or dedicated coverage of an athletic event, having an advanced level provider (i.e., advanced EMT or paramedic) is recommended to ensure timely access to advanced care, especially if there is no known medical coverage on site.

Strengths and limitations

A strength of this study was the use of an operational definition for a sport-related injury that included both causes of injury and incident location type variables. Agency participation in the NEMSIS is voluntary; thus, the database relies on a convenience sample of EMS agencies across the US and does not include all states and territories. Large proportions of "not applicable" or "not reported" data were excluded from analyses. Each EMS activation represents an event documented by a responding EMS agency; therefore, multiple EMS activations can have occurred for a single patient. Future research is warranted to investigate how access to on-site medical care and appropriate emergency planning may affect EMS utilization for sport-related injuries. Additional research is also warranted to examine outcomes (e.g., ED care provided, hospital disposition, re-contact of EMS) of patients for whom a sport-related EMS activation occurred. The ability to link data from EMS agencies to hospitals would provide valuable insight into the accuracy of EMS provider impressions and care provided and aid in determining medical necessity to inform prehospital care decisions better.

## Conclusions

This is the first study to examine sport-related injuries encountered by EMS using a standardized national database in the United States. Considering that not all EMS agencies are required to report to the NEMSIS, the number of EMS activations for sport-related injuries is likely higher than reported in this study. Most EMS activations for sport-related injuries resulted in transportation by EMS and receiving an advanced level of care, indicating the EMS activation was likely necessary. Emergency medical services agencies providing medical coverage for athletic events should consider providing an ALS-level unit when resources allow. Planning for athletic events should be conducted in coordination with local EMS agencies, especially for larger athletic events that may strain local medical resources should a large-scale emergency occur. Sport-related injuries resulting in EMS activations differ between the adult and pediatric populations, warranting additional research into how sport-related injuries, particularly those emergent in nature, may change across the lifespan.
